# Hypertrophic Pulmonary Osteoarthropathy: A Case Report

**DOI:** 10.4021/wjon550w

**Published:** 2012-10-28

**Authors:** Eric Royston, Khalil Katato, Dan Mesko

**Affiliations:** a Genesys Regional Medical Center,One Genesys Parkway,Grand Blanc, MI 48439, USA; b Genesee Hematology Oncology, 302 Kensington Ave, Flint, MI 48503, USA

**Keywords:** Hypertrophic pulmonary osteoarthropathy, Sarcomatoid carcinoma of the lung

## Abstract

A 42-year-old male presented to the emergency department with difficulty breathing and body pain particularly in his bilateral lower extremities. A workup was done and he was diagnosed with sarcomatoid carcinoma of the lung. Bone scintigraphy was performed and hypertrophic pulmonary osteoarthropathy was diagnosed to be the source of the pain.

## Introduction

Hypertrophic Pulmonary Osteoarthropathy (HPO) is a subcategory of hypertrophic osteoarthropathy (HOA). HOA is characterized by clubbed fingers, periostitis of long bones and arthritic changes [1]. HPO is an arthropathy that results from a pulmonary disease [1]. HPO has been shown to be associated with lung neoplasms, congenital cyanotic heart disease, liver disease and other systemic diseases that have pulmonary effects [2]. The most common, however, is that of intrathoracic neoplasms which accounts for approximately 90% of HPO cases and of those 80% are lung cancer [1]. Although HPO is most commonly seen in lung neoplasms, the overall incidence of HPO in lung cancer patients is very low. According to one study, less than 1% of lung cancer patients develop HPO as a paraneoplastic syndrome while historical studies have published anywhere from 0.2-17% [1]. The demographics of the lung cancer patients that most commonly develop HPO are males, heavy smokers and advanced disease [1].

## Case Report

**Figure 1:**
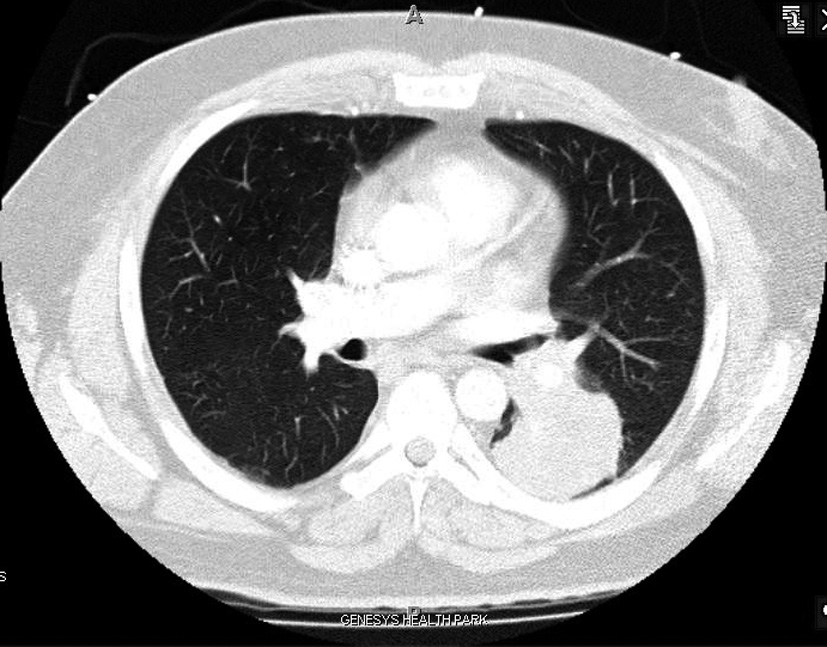
Sarcomatoid Carcinoma located in LLL on CT Angio.

**Figure 2:**
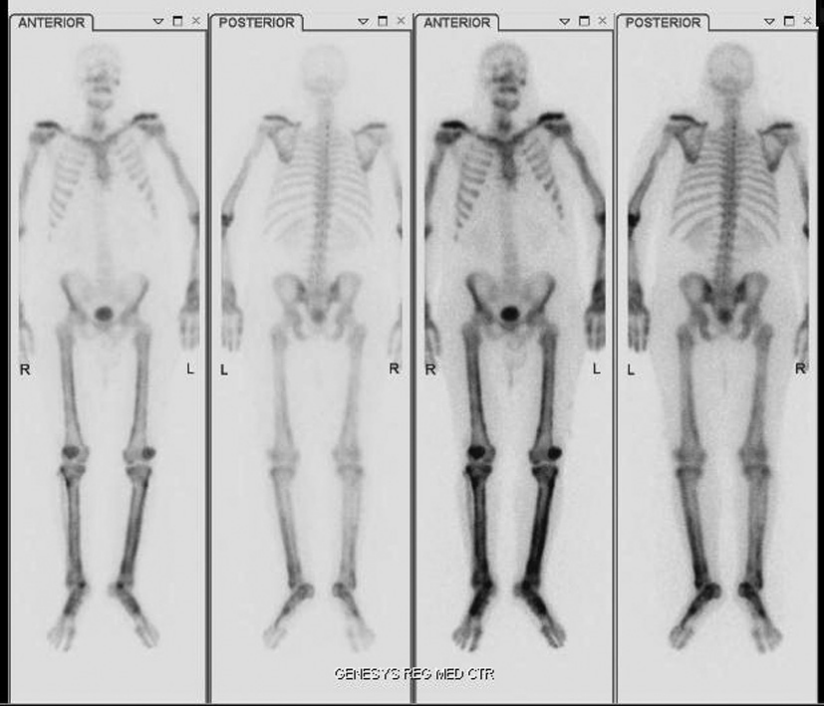
HPOA demonstrated on Bone Scan with increased peripheral periosteal and cortical uptake of technetium-99m MDP in the femoral and tibial bones bilaterally as well as clavicles and midfoot.

A 42 year old Caucasian male with no history of smoking and with a past medical history significant for hypertension and hypercholesterolemia presented to the emergency department (ED) with complaints of worsening shortness of breath over the past month. Upon further examination the patient complained of a 2 - 3 month history of bilateral upper and lower extremity pain, a 50 pound weight loss over 5 months, night sweats and persistent fever and chills. A computed tomography angiogram was ordered in the ED due to the suspicion of pulmonary embolism which was subsequently negative for pulmonary embolism (PE), but a 5.5 x 5.0 cm mass was noted in the left lower lobe that encased the bronchus and pulmonary artery (Fig. 1). Computed tomography (CT) guided biopsy was performed and the subsequent pathology report described findings consistent with sarcomatoid carcinoma. While awaiting pathology of the lung mass, further examination into the extremity pain was worked up and a bone scan was performed (Fig. 2) On bone scintigraphy there is increased peripheral periosteal and cortical uptake of technetium-99m MDP bilaterally in the femurs, tibias, clavicles and midfeet demonstrating the unique characteristics that are consistent with hypertrophic pulmonary osteoarthropathy secondary to the lung cancer. The patient underwent a pneumonectomy via muscle sparing thoracotomy and after recovering from ventilator dependent respiratory failure, secondary to acute respiratory distress syndrome, the patient began to demonstrate improvement in his symptoms. The pain in his legs started to subside and the patient is schedule to follow up in the clinic to begin chemotherapy.

## Discussion

The aforementioned male patient, although a non-smoker and relatively young, developed a unique lung cancer diagnosed as sarcomatoid carcinoma of the lung. The pain that he was experiencing was worked up and found to be HPO. The following information is specific to the characteristics of HPO.

### Pathogenesis

The pathogenesis of HPO is not well understood. Several hypotheses exist but no conclusive studies demonstrate a definitive answer. One theory states that there needs to be arteriovenous shunts present for HPO to occur [2]. Megakaryocytes are in the pulmonary capillary bed and release factors such as vascular endothelial growth factor and platelet derived growth factors that induce the changes seen in HPO [1, 2]. This theory is backed by patients who develop later metastasis in non-pulmonary locations who do not have relapse of HPO [2].

### Imaging

Initial imaging is x-ray. Periosteal membrane thickening is the hallmark of HPO and can be visible on plain radiograph. Bone scintigraphy is a helpful imaging tool that can also be used. On bone scintigraphy there will be increased peripheral periosteal and cortical uptake of technetium-99m typically in the long bones.

### Treatment

There is no true treatment of HPO. In one study, treatment of the cancer itself led to relief of symptoms and improved bone scintigraphy in half of the patients [1]. In a series of two case reports, one demonstrated that one patient had complete relief of symptoms following chemotherapy [3]. Symptomatic treatment can include NSAIDs, bisphosphonates, or octreotide [1].

### Conclusion

Hypertrophic Pulmonary Osteoarthropathy is a subset of Hypertrophic Osteoarthropathy that is most commonly associated as a paraneoplastic syndrome of lung cancer. The etiology of HPO is unknown; however, symptoms have shown to resolve in over half of patients with treatment of the underlying cause. Symptomatic treatment can be used until HPO is resolved.

## Author Notes

Eric Royston and Dan Mesko were medical students rotating on a Hematology/Oncology rotation with Dr. Khalil Katato while at Genesys Hospital. Eric Royston is currently an intern at Tripler Army Medical Center in Honolulu, HI. Dan Mesko is currently an Orthopedic Intern at The Cleveland Clinic.
